# Collection on groundwater to celebrate the World Water Day March 20, 2022

**DOI:** 10.1016/j.mex.2022.101648

**Published:** 2022-02-23

**Authors:** D. Barcelo

**Affiliations:** a Department of Environmental Chemistry, Institute of Environmental Assessment and Water Research, (IDAEA-CSIC), Jordi Girona 18-26, Barcelona 08034, Spain; b Catalan Institute for Water Research, ICRA-CERCA,H2O Building, Emili Grahit, 101, 17003 Girona, Spain

I am delighted to announce this new Methods X Article Collection on Groundwater on the occasion of the World Water Day 2022 dedicated this year to highlight the importance of this water resource.

Groundwater constitutes about 30% of the world's freshwater resources. From the other 70% nearly 69% is captured in the ice caps and mountain snow and glaciers and only 1% is located in rivers and lakes. The thing is that according to these figures, groundwater is one of the keys to sustainability of world water resources and there is an urgent need to apply better management practices. Groundwater is extensively used worldwide for domestic, industrial, and agricultural purposes. In this sense both urban and rural areas rely on groundwater resources to meet their water demands. Over-exploitation of groundwater resources reduces water availability and results in negative impacts on groundwater-dependent ecosystems, such as springs and wetlands. In short, the main negative impacts to groundwater quality are caused by saline intrusion, the entrance of chemicals by diffuse sources, i.e., agricultural practices, by point sources such as the leaking of city sewage pipelines and lastly by inefficient waste management practices originated in landfills and other industrial activities like mining.

Based on everything has been said groundwater quality and quantity issues are the major challenges identified and are discussed in the twelve papers selected in this article collection. These papers were published in the last few years in Methods X and evaluate practical aspects and state of the art methodologies such as:

- Conceptual model to include a broad range of emerging contaminants groundwater monitoring programs
- Advances in mass spectrometric instrumentation to determine emerging organic contaminants and arsenic in groundwaters
- Health risk assessment of nitrate leachates from agriculture and its impact on groundwater resources in mountainous catchments and as a source for drinking water
- Hazard assessment models to predict and manage the impact of different types of landfills in groundwater resources
- Water quantity and hydrological investigations using numerical and mechanistic models to investigate the groundwater potential zone of the aquifers
- River and groundwater model algorithms for simulating the response of river water temperature to urban greening

Examples reported here describe many different aspects related to groundwater research challenges. The first example discusses the need to develop a conceptual model that integrates various monitoring programs covering a broad range of organic wastewater contaminants like pesticides, pharmaceuticals and personal care products among others. The following two papers report mass spectrometric (MS) methods such as the use of liquid chromatography-MS coupled to solid phase extraction for the analysis of ubiquitous pesticides like atrazine and metolachlor or the application of inductively coupled plasma-MS for the determination of arsenic species. Nitrate contamination of groundwaters is a global problem not only in catchment level but also for drinking water purposes and it also part of this article collection. A drinking water quality index that includes nitrates and other anions and cations has been proposed in one of the papers selected. The impact of landfill leachate plumes on ground water has been in the environmental agenda for quite some time. Examples reported here include industrial and municipal waste and sanitary landfills as well as models for landfills selection sites according to a comprehensive list of criteria such as land use, protected area, surface water and groundwater resources in the potential landfill location. The last examples reported in this collection are related to water quantity and hydrological investigations. Needless to say that stochastic and mechanical models are generally used to predict groundwater availability considering aquifer recharge and evapotranspiration among other variables. Location of groundwater potential zones is of great importance in order to unequivocally identify the quantity of ground water resources in certain geographical area. A river and groundwater model algorithm that captures the warming and cooling impact of urban development and restoration through a water and energy budget is described in the last paper of this article collection.

That being said I would like to thank all authors of these twelve contributions to Methods X in compiling such a timely article collection. Needless to say that this groundwater collection papers will be of help to environmental scientists, specially hydrologists as well as newcomers, PhD students and those senior researchers who consider groundwater as one of the world resources that needs protection.

Ultimately I have the pleasure to announce a Virtual Special Issue on Groundwater to celebrate this World Water Day 2022. This will allow authors who had not the opportunity to be part of this VSI that is now released as well as to previous authors who contributed to this Methods X article collection to update and publish their recent methods. Submission window will be open from March 20 till October 31st 2022.

As always, thanks for reading and stay safe and healthy!

